# Dental Neglect Among Children in Eastern Turkey

**DOI:** 10.3290/j.ohpd.c_1834

**Published:** 2025-02-20

**Authors:** Peris Celikel, Aybike Bas Ozturk, Fatih Sengul

**Affiliations:** a Peris Celikel Assistant Professor Department of Pedodontics, Ataturk University, Faculty of Dentistry, Erzurum, Turkey. Concept and design, investigation, visualisation, wrote original draft, reviewed and edited the manuscript.; b Aybike Bas Ozturk Assistant Professor, Department of Pedodontics, Ataturk University, Faculty of Dentistry, Erzurum, Turkey. Concept and design, data curation, visualisation, reviewed and edited the manuscript.; c Fatih Sengul Associate Professor, Department of Pedodontics, Ataturk University, Faculty of Dentistry, Erzurum, Turkey. Concept and design, methodology, wrote original draft, reviewed and edited the manuscript.

**Keywords:** Dental Neglect Scale, DMFT, parents

## Abstract

**Purpose:**

Dental neglect is defined as the failure of parents to take necessary measures to protect their child’s oral health, prevent pain and infection, and provide essential dental treatment. This study aims to assess the level of dental neglect among children in Eastern Turkey and its relation to their oral health.

**Materials and Methods:**

This cross-sectional study involved the 215 children and their parents. Dental neglect was measured using the Dental Neglect Scale (DNS), which consisted of seven Likert-scale questions (ranging from 1 to 5). A questionnaire also gathered demographic data. The DNS score was calculated by summing the responses, and the children’s oral health was assessed using the Oral Hygiene Index and the Decayed, Missing, and Filled Teeth (DMFT) index. Data were statistically analysed with significance set at p < 0.05.

**Results:**

The average DMFT score was 7.24 ± 3.01, and the average DNS score was 16.24 ± 4.72. A statistically significant correlation was found between DMFT and the DNS score (correlation coefficient 0.162, p = 0.018). A statistically significant difference was noted between the mother’s education level and the Dental Neglect Score (p = 0.006), but no statistically significant differences were observed concerning paternal education, family income, or frequency of dental visits.

**Conclusion:**

High DMFT scores and low maternal education levels are linked to higher dental neglect. Mothers play a crucial role in their children’s oral health. Identifying mothers who do not provide adequate attention to their children as well as children in need of oral care is essential for implementing early, tailored interventions.

Oral health is an integral part of overall health and plays a crucial role in an individual’s well-being.^10^ Genetic factors, hormonal changes, bad habits, diet, brushing habits, and personal behaviours and attitudes have a role in oral health.^15,22^ Dental neglect is defined as the failure of parents to take measures to alleviate children’s dental pain and infection and to provide necessary dental treatment to protect oral health.^4^

Dental neglect can occur at every stage of life due to various underlying reasons. Although dental neglect may seem like an isolated issue in children, it can actually be an indicator of other forms of abuse.^9^ Dental neglect in children leads to dental pain, difficulty eating, weight loss, infection, loss of function, sleep disorders, aesthetic problems, poor performance at school, low self-esteem, and ultimately, reduced quality of life.^4,15^ In addition, psychological, emotional, and social negative effects that impact the child’s overall well-being may also emerge in the long term.^17^

The Dental Neglect Scale evaluates how much attention an individual gives to their teeth, whether they seek professional dental care, and their belief in the significance of oral health.^7^ This seven-item scale was developed to assess parents’ behaviours and attitudes towards their children’s oral health. The neglect of parents responsible for meeting their children’s health needs has led to higher dental neglect scores, and it has been reported that these children have more cavities and fewer dental visits.^23^

Many studies have been conducted on dental neglect in different populations around the world.^4,17^ However, new studies on dental neglect among children of different races and age groups are important for gaining new insights about these children. This information can help us address and assist children at risk. Additionally, investigating dental neglect in children will identify the specific reasons for inadequacies in preventing or treating dental caries. This will provide the government and healthcare professionals with the opportunity to address the issue comprehensively. Therefore, the aim of our study was to assess the level of dental neglect in children with high caries incidence in Eastern Turkey^21^ and to determine the relationship between dental neglect and the children’s oral health status. The null hypothesis of our study is that there is no relationship between the level of dental neglect and the oral health status of children in Eastern Turkey.

## MATERIALS AND METHODS

Our study protocol was found to be in accordance with local regulations and the principles of the Declaration of Helsinki, and received approval from the local Ethics Committee of Atatürk University Faculty of Medicine (2/31, 29.03.2024). In the Pearson correlation test, to achieve statistical power of 80% with a minimum effect size of 0.4 and a significance level of 0.05, the required number of participants was found to be 204. Considering possible losses, 215 parents of children who applied to the Department of Pediatric Dentistry at Atatürk University Faculty of Dentistry were included in this cross-sectional study. Written informed consent was obtained from the parents of all participants in the study.

Parents and children who volunteered to participate in the study and had no cooperation or emotional issues were included. Patients with any acute or chronic systemic or immune-related conditions, syndromic or congenital anomalies, or whose guardians declined participation were excluded from the study.

Parents were asked to fill out a questionnaire that included questions about demographic characteristics, the child’s history of trauma, the presence of bad oral habits, and visits to the dentist. Additionally, parents completed a questionnaire investigating the Dental Neglect Scale (DNS), which consisted of seven questions using a Likert scale ranging from one (“strongly disagree”) to five (“strongly agree”).^23^ The DNS score was calculated by summing the scores of the seven questions, with scores ranging from 7 to 35; higher scores indicate greater dental neglect.

The oral health status of the children was clinically assessed using the decayed, missing, and filled teeth (DMFT) index.^27^ The oral examinations of the children were performed by two dentists. The children’s oral examinations were conducted in natural daylight using a disposable mouth mirror and conventional dental probes.

To determine inter-observer variability of DMFT, evaluations were repeated on 30 patients after 3 weeks.

### Statistical Method

Descriptive statistics (count, percentage, mean, standard deviation, minimum, maximum, and median) of the data were provided in the study. The normality assumption was assessed using the Shapiro-Wilk test, and the homogeneity of variance was evaluated with Levene’s test. ANOVA was used to compare the means of three or more independent groups with a normal distribution, while the Kruskal-Wallis test was applied for non-normal distributions. Spearman’s correlation measured the relationship between continuous variables that did not follow a normal distribution, and Kendall’s Tau correlation was used to assess the relationship between ordinal categorical and continuous variables. Pearson’s chi-squared test was employed to test the relationship between categorical variables when the sample size assumption (expected value > 5) was met, while Fisher’s Exact test was used when this assumption was not satisfied. Inter-rater reliability for DMFT measurements was evaluated using the intraclass correlation coefficient and kappa coefficient. Analyses were conducted using IBM SPSS version 25, with statistical significance set at p < 0.05.

## RESULTS

The demographic characteristics of the study groups and the distribution of responses to the survey questions are presented in Table 1. A total of 215 children aged between 4 and 15 years (mean age 8.8 ± 2.6 years) participated in the study, with 109 girls (50.7%) and 106 boys (49.3%). It was determined that half of the participants had “more than the minimum wage” income. 23.3% of the children had bad oral habits. Examining the frequency of dental visits over the past two years, it was found that 7.9% of the children never visited a dentist and 43.3% only visited when they experienced pain.

**Table 1:** Distribution of participants’ demographic characteristics and responses to survey questions

Gender	Female	109	50.7
Male	106	49.3
Mother’s education level	Primary school	97	45.1
Middle school	36	16.7
High school	54	25.1
University	28	13.0
Father’s education level	Primary school	40	18.6
Middle school	29	13.5
High school	79	36.7
University	67	31.2
Monthly income	Less than the minimum wage	36	16.7
Minimum wage	71	33.0
More than the minimum wage	108	50.2
Does your child have any illness?	Yes	11	5.1
No	204	94.9
Is your child currently taking medication?	Yes	10	4.7
No	205	95.3
Has your child previously experienced trauma?	Yes	28	13.0
No	187	87.0
Does your child have any bad habits?	No	165	76.7
Teeth grinding	20	9.3
Thumb sucking	15	7.0
Pencil biting	9	4.2
Mouth breathing	6	2.8
Frequency of dental visits in the last two years	6 months to 1 year	105	48.8
When experiencing pain	93	43.3
Never visited	17	7.9

**Fig 1:**
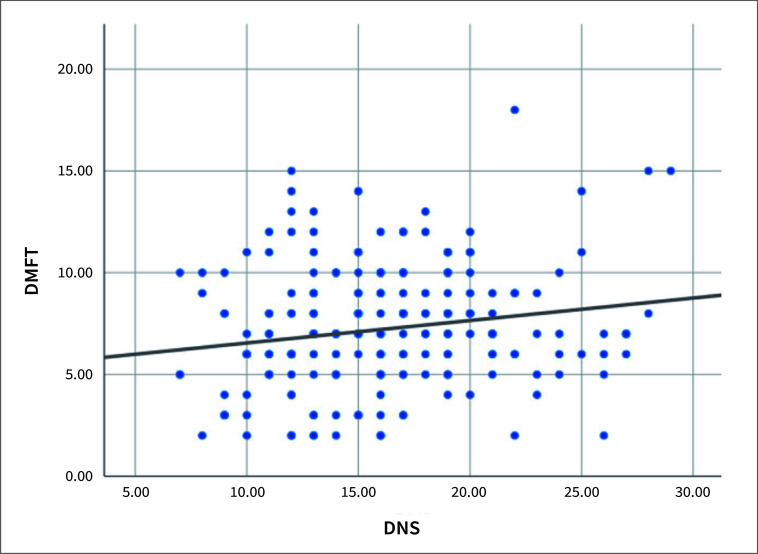
Scatter plot of the distribution of children’s DMFT and DNS scores.

There was high concordance between researchers for DMFT measurements (Kappa statistics: 0.96–0.93; inter-rater correlation: 92%). Descriptive statistics of individuals’ DMFT and DNS scores are given in Table 2, and the scatter plot is illustrated in Fig 1. The averages for DMFT and DNS were found to be 7.24 ± 3.01 and 16.24 ± 4.72, respectively. A statistically significant relationship was observed between DMFT and DNS with a correlation coefficient of 0.162 (p=0.018). An increase in DMFT scores is associated with a corresponding increase in DNS scores.

**Table 2:** Distribution and correlation of participants’ DMFT and dental neglect scale scores

DMFT	7.24	3.01	0.162	0.018
DNS	16.24	4.72		

Descriptive statistics of the distributions of parents’ responses to the DNS questions are presented in Table 3. For questions related to the child’s ability to maintain dental hygiene and their opinion on the importance of dental health, respondents expressed “Disagree” more frequently. Similarly, for the question “Needs dental care” which was answered separately by both the parents and the child, the response was also “Disagree” more often. In contrast, the answer “Agree” was given more frequently to the question of whether child can control meal snacking.

**Table 3:** Distributions of parents’ responses to the Dental Neglect Scale questions

1. Maintains his/her dental care	Strongly disagree	54	25.1
Disagree	87	40.5
Neutral	36	16.7
Agree	28	13.0
Strongly agree	10	4.7
2. Your child performs oral care at home	Strongly Disagree	94	43.7
Disagree	95	44.2
Neutral	17	7.9
Agree	6	2.8
Strongly agree	3	1.4
3. Needs dental care: parent postpones*	Strongly disagree	91	42.3
Disagree	69	32.1
Neutral	10	4.7
Agree	33	15.3
Strongly agree	12	5.6
4. Needs dental care: child postpones*	Strongly disagree	102	47.4
Disagree	62	28.8
Neutral	4	1.9
Agree	25	11.6
Strongly agree	22	10.2
5. Brushes as well as he/she should	Strongly disagree	47	21.9
Disagree	69	32.1
Neutral	43	20.0
Agree	37	17.2
Strongly agree	19	8.8
6. Controls between-meal snacking	Strongly disagree	17	7.9
Disagree	56	26.0
Neutral	37	17.2
Agree	59	27.4
Strongly agree	46	21.4
7. Considers dental health important	Strongly disagree	69	32.1
Disagree	93	43.3
Neutral	22	10.2
Agree	19	8.8
Strongly agree	12	5.6

Descriptive statistics for the distributions of DNS scores according to participants’ demographic characteristics are presented in Table 4. A statistically significant difference in DNS scores was found based on the mother’s educational level (p=0.006). The difference in DNS scores between mothers with only elementary education and those with university education was statistically significant, with elementary-educated mothers having higher scores. There were no statistically significant differences in DNS scores based on father’s education level, income level, and frequency of dental visits in the past two years (p > 0.05).

**Table 4:** Distribution and comparison of DNS scores by participants’ demographic characteristics

Mother’s educational status	Primary school	17.42 ± 5.05^a^	0.006*
Middle school	15.56 ± 4.22 ^a,b^	
High school	15.76 ± 4.48^a,b^	
University	13.93 ± 3.46^b^	
Father’s educational status	Primary school	17.00 ± 4.03	0.051
Middle school	17.45 ± 5.23	
High school	16.46 ± 4.78	
University	15.00 ± 4.64	
Income	Less than minimum wage	17.53 ± 4.46	0.146
Minimum wage	15.63 ± 5.10	
More than minimum wage	16.20 ± 4.50	
How often has your child visited the dentist in the last two years?	6 months-1 year	15.93 ± 4.65	0.524
In the presence of pain	16.40 ± 4.84	
Never	17.24 ± 4.62	

## DISCUSSION

Dental neglect in children refers to situations where, despite having adequate access to dental services, parents or caregivers fail to sufficiently prioritise the child’s dental health and intentionally neglect to seek or follow up on necessary treatments to protect the child from pain and infection.^9,10^ This neglect is associated with factors such as inadequate toothbrushing habits, parents not taking the child to regular dental visits, and poor nutritional habits. As a result, children may develop caries, periodontal diseases, and other oral health problems.^3,14,18^ Therefore, dental neglect is an important issue with a high prevalence. In this study, it was observed that the average DNS score was high at 16.24 ± 4.72, indicating that dental neglect is a concern in Eastern Turkey and needs to be addressed. Managing this issue is crucial for both psychological and physical health policies. There is limited literature worldwide on identifying dental neglect in children. According to the results of our study, an increase in DMFT and a decrease in the mother’s education level were associated with an increase in the DNS score. These findings are contrary to our null hypothesis. To the best of our knowledge, this study is the first in the literature to address dental neglect among children in Eastern Turkey.

A review of the literature on studies on dental neglect yielded the following: Montecchi^16^ found that children who experienced maltreatment had significantly higher rates of caries, plaque accumulation, and gingival bleeding than those in the control group. Thomson and Gaughwin^23^ identified a positive correlation between dental neglect and children who had not received dental treatment in the past two years, particularly in families with low socioeconomic status. This latter characteristic was also noted in two additional studies. Butts and Henderson^6^ indicated that indicators such as untreated cavities, pain from infections, bleeding, orofacial injuries, and a record of inconsistent care should be used to identify dental neglect in children. Fakhruddin et al^8^ examined the social impact of untreated dental issues in children and reported that those with untreated dental trauma experienced more difficulty in chewing, avoided smiling, and faced challenges in social interactions. In this study, it was observed that as the prevalence of caries increased and the mother’s educational level decreased, dental neglect also rose. Based on studies in the literature and the findings of this study, it is evident that dental neglect is a serious issue that negatively impacts children’s quality of life. It is crucial to eliminate the factors contributing to dental neglect and to find solutions to this problem.

Dental caries, the most common multifactorial chronic disease in children worldwide, is an indicator of dental neglect.^13,17,25^ Factors contributing to caries in children include parental neglect of the child’s nutrition, missed dental visits, and failure to instill proper oral hygiene habits. As a result of dental neglect leading to caries, children may experience short-term complications such as pain and oral infections, as well as long-term issues like tooth loss, oral abscesses, widespread infections, sleep deprivation, and reduced body weight due to eating problems.^2,11^ The literature indicates that an increase in the number of cavities correlates with a higher DNS score.^7,10,28^ Our study’s results align with these findings, showing that dental neglect leads to a deterioration in children’s oral health. Given this, it is suggested that the high incidence of caries in Eastern Turkey may be a consequence of dental neglect.

Low educational level is among the factors contributing to dental neglect. It has been found that individuals with lower educational levels, lower occupational status, and lower socioeconomic status exhibit increased dental neglect.^1,14,20^ Our study findings indicate that the father’s educational level does not affect the DNS score. Additionally, as the mother’s educational level decreases, the DNS score increases. Mothers with higher education levels generally place more importance on their children’s dental health and assist in developing their oral hygiene habits. Therefore, it is likely that mothers with higher education levels have achieved lower DNS scores for their children. Based on the results of our study, it appears that mothers play a primary role in the oral health of children in Eastern Turkey. To reduce DNS scores, it may be beneficial to implement educational programs aimed at increasing mothers’ knowledge and awareness about oral and dental health.

The family’s socioeconomic status is among the factors that significantly affect oral health.^26^ The literature indicates that lower-income families tend to have higher DNS scores.^8,26^ However, our study observed that income level did not affect the DNS. These results suggest that dental neglect is more closely related to educational level than to income level.

Parental indifference towards acquiring information about dental care, failure to perform dental care at home, and not taking children to dental appointments are factors contributing to dental neglect in children.^5,19^ A study conducted in Brazil on dental neglect indicated that parents’ failure to take their child to the dentist because they believe it is unnecessary places them at risk for dental neglect.^15^ In contrast, a study conducted in Greece reported that 95% of parents believed their child needed to visit the dentist at an early age, and 79.5% of respondents stated that they had previously taken their child to the dentist for various reasons.^12^ In our study, an examination of the frequency of dental visits over the past two years showed that 7.9% of the children had never visited a dentist, 48.8% had visited within 6 months to 1 year, and 43.3% had visited only in the presence of pain. Despite being in an area with a publicly available (free) oral health system, our data reveal that a statistically significant portion of patients only visited the dentist when experiencing pain. The establishment of family dental centers is crucial to ensure that parents are not only encouraged to bring their children for routine dental check-ups but also educated about preventive measures and provided with the necessary guidance.

When interpreting the findings of this study, several limitations must be considered, including the restricted geographic scope of the observations. A multi-center study in Turkey could provide a more comprehensive understanding of dental neglect among Turkish children. Another limitation is that our study’s results relied solely on self-reported data from parents.

## CONCLUSION

Dental neglect is prevalent among children in Eastern Turkey. An increase in caries severity has been shown to correlate with a higher dental neglect score. The mother’s level of education plays a significant role in the child’s dental care knowledge and attitudes. Therefore, educational programs are needed to enhance parents’ knowledge, understanding, and attitudes regarding oral health.
